# Early life environment and natural history of inflammatory bowel diseases

**DOI:** 10.1186/s12876-014-0216-8

**Published:** 2014-12-16

**Authors:** Abra Y Guo, Betsy W Stevens, Robin G Wilson, Caitlin N Russell, Melissa A Cohen, Holly C Sturgeon, Anna Thornton, Cosmas Giallourakis, Hamed Khalili, Deanna D Nguyen, Jenny Sauk, Vijay Yajnik, Ramnik J Xavier, Ashwin N Ananthakrishnan

**Affiliations:** Division of Gastroenterology, Massachusetts General Hospital, Boston, MA USA; Harvard Medical School, Boston, MA USA; Massachusetts General Hospital Crohn’s and Colitis Center, 165 Cambridge Street, 9th Floor, Boston, MA 02114 USA

**Keywords:** Breastfeeding, Early life, Microbiome, Crohn’s disease, Surgery

## Abstract

**Background:**

Early life exposures may modify risk of inflammatory bowel diseases (IBD; Crohn’s disease (CD), ulcerative colitis (UC)). However, the relationship between early life exposures and natural history of IBD has not been previously examined.

**Methods:**

This single center study included patients with CD or UC recruited in a prospective IBD registry. Enrolled patients completed a detailed environmental questionnaire that assessed various early life environmental exposures. Our primary outcome was requirement for disease-related surgery in CD and UC. Logistic regression models defined independent effect of early life exposures, adjusting for potential confounders.

**Results:**

Our study included 333 CD and 270 UC patients. Just over half were female with a median age at diagnosis of 25 years. One-third of the cohort had history of bowel surgery (31%) and nearly half had used at least one biologic agent (47%). Among those with CD, being breastfed was associated with reduced risk of CD-related surgery (34% vs. 55%), while childhood cigarette smoke exposure was associated with increased risk. On multivariate analysis, history of being breastfed (odds ratio (OR) 0.21, 95% confidence interval [CI] 0.09–0.46) and cigarette smoke exposure as a child (OR 2.17, 95% CI 1.10–4.29) remained independently associated with surgery. None of the early life variables influenced disease phenotype or outcome in UC.

**Conclusion:**

A history of being breastfed was associated with a decreased risk while childhood cigarette smoke exposure was associated with an increased risk of surgery in patients with CD. Further investigation to examine biological mechanisms is warranted.

**Electronic supplementary material:**

The online version of this article (doi:10.1186/s12876-014-0216-8) contains supplementary material, which is available to authorized users.

## Background

Inflammatory bowel disease (IBD), which comprises Crohn’s disease (CD) and ulcerative colitis (UC), are chronic immune mediated diseases characterized by a dysregulated immune response to commensal flora in a genetically susceptible host [1]. The expanded set of 163 distinct risk alleles for CD and UC together explain less than one-third of the heritability of disease [2]. The relatively recent secular increase in disease incidence [3] and the changes in disease risk occurring with migration [4,5] suggest an important role for the modifiable external environment in pathogenesis. Growing literature demonstrating microbial dysbiosis in patients with IBD with reduced diversity [1,6-8], decreased representation of potentially protective bacteria like *Faecalibacterium prausnitzii* [9] and increased frequency of potentially pathogenic bacteria in certain disease subsets like ileal CD [10] suggests that one possible mechanism through which the environment influences disease risk may be through the microbiome.

Given the peak age of incidence of IBD in childhood and early adulthood, the role of early life environmental factors is of considerable interest. The spectrum of risk factors examined range from gestational age and mode of delivery to breastfeeding, antibiotic use, exposure to farm animals or pets, and cigarette smoke, all of which have variably been demonstrated to modify risk of CD or UC [11-19]. It is of note that few studies have examined whether such early life environmental exposures modify natural history of disease by influencing disease phenotype or severity. Given the growing data on the role of microbiome in determining natural history of disease, it is indeed plausible that such early life exposures may influence disease phenotype and outcomes beyond incident disease. A particularly promising such factor is breastfeeding. Breastfeeding is known to have protective benefits for infants due to the immunomodulatory properties of breast milk [20-22] and the effect of human milk oligosaccharides on the infant microbiota. Furthermore, secretory antibodies in breast milk promote intestinal homeostasis, regulate gut microbial composition, and modulate host gene expression [20-22].

Examining the influence of these early life environmental factors on disease outcome could shed light on its interplay with microbiome, potentially leading to novel therapies targeting intestinal flora. Consequently, we performed this study using a large, well-phenotyped cohort of IBD patients to investigate the effects of early environmental exposures on disease course in both CD and UC.

## Methods

### Study population

The study population consisted of adult patients enrolled in the Prospective Registry in IBD Study at Massachusetts General Hospital (PRISM). This is a single center ongoing patient registry that aims to understand the causes of IBD and factors affecting disease progression. Patients were approached during their regularly scheduled clinic visits at the MGH Crohn’s and Colitis Center. Study research coordinators obtained consent and medical history was obtained and confirmed by review of the electronic medical record.

### Variables

Environmental questionnaires including ten questions on early life exposures were distributed to patients during regularly scheduled clinic visits subsequent to enrollment. The first wave of questionnaires was distributed in 2010 (n = 348) and the second wave to an additional set of patients was distributed during January-June 2014 (n = 255). Early life exposures included questions on gestational age, history of breastfeeding, exposure to cigarette smoke in childhood, treatment with antibiotics, hospitalization, and exposure to pets or farm animals (Additional file 1). Those who answered “Not sure” for any of the exposures were treated as missing for that variable.

### Covariates and outcomes

Information on age at diagnosis, demographics, family history of IBD, and disease behavior, location or extent was obtained during the study visit. Age at diagnosis was assessed as <16 years (A1), 17 to 40 years (A2), >40 years (A3) according to the Montreal classification [23]. Disease location was characterized as terminal ileum (L1), colon (L2), ileocolon (L3) or upper gastrointestinal (L4) disease. In CD, behavior was characterized as inflammatory (B1), stricturing (B2), and penetrating (B3), each with the potential modifier of perianal involvement. Disease extent in UC was characterized as proctitis (E1), left-sided colitis (E2), pancolitis (E3).

Our primary outcome was IBD-related bowel resection for both CD and UC while our secondary outcome included need for anti-tumor necrosis factor biologic therapy (infliximab, adalimumab, certolizumab pegol). IBD-related surgery was selected as our primary outcome as this is a more robust and hard endpoint that is less influenced by physician practice variation. Disease characteristics and outcomes were confirmed by the treating physician.

### Statistical analysis

The data was analyzed using Stata 13.0 (StataCorp, College Station, TX). Separate analyses were conducted for CD and UC. Patients with indeterminate colitis were grouped with UC owing to small numbers of the former. Continuous variables were summarized using means and standard deviations while categorical variables were expressed as proportions and compared using the chi-square test. We first performed univariate analysis comparing the environmental risk factors to our primary and secondary outcomes of interest. Multivariate regression models adjusting for disease specific covariates including phenotype and duration since diagnosis were used to identify the independent association of the exposures with our outcome of interest. This also allowed for exploration of whether the early life environmental influences exerted their effect through modification of disease phenotype. A p-value of <0.05 was considered statistically significant. Ethical approval for this study was obtained from the Institutional Review Board of Partners Healthcare (2004P001067). All patients enrolling in PRISM provided informed consent for participation in the registry.

## Results

A total of 603 patients completed questionnaires on early life exposures (333 CD; 270 UC/IC). Just over half the cohort was female (54%), and 23% had family history of IBD in a first-degree relative. The median age at diagnosis of IBD was 25 years (interquartile range (IQR) 18 – 35 years) with median disease duration of 9 years at the time of enrollment (IQR 4 – 16 years). Nearly half of those with CD had inflammatory disease (44%), a third had penetrating disease (36%) while the remainder had stricturing phenotype. The most common location was ileocolonic (51%) followed by colon alone (24%) or ileal alone (18%). Over half of those with UC (57%) had pancolitis. In the entire cohort, 186 patients (31%) had at least one IBD-related surgery, two-thirds had used immunomodulators (65%) and half required biologics (46%). Table 1 describes the frequency of occurrence of various environmental exposures. Just over half the cohort had a history of being breastfed in infancy. Few were delivered via Caesarean section, required hospitalization in childhood, or grew up on a farm.

**Table 1:** **Characteristics of the study population: frequency of environmental exposures**

**Environmental exposure**	**Crohn’s disease**	**Ulcerative colitis**
	**N (%)**	**N (%)**
**Breastfeeding**		
No	124 [46]	81 (45)
Yes	146 (54)	101 (55)
**Delivery via caesarean section**		
No	258 (86)	172 (84)
Yes	41 (14)	34 (16)
**Hospitalization before age five years**		
No	217 (78)	158 (82)
Yes	60 (22)	35 (18)
**Childhood pets**		
No	102 (33)	75 (33)
Yes	208 (67)	151 (67)
**Growing up on farm**		
No	299 (97)	215 (96)
Yes	10 (3)	10 (4)
**Exposure to daycare during childhood**		
No	178 (60)	131 (61)
Yes	121 (40)	84 (39)
**Childhood exposure to cigarette smoke**		
No	140 (47)	157 (53)
Yes	113 (52)	104 (48)

On univariate analysis, a history of being breastfed was associated with reduced risk of surgery in CD (odds ratio (OR) 0.42, 95% confidence interval (CI) 0.25 – 0.68) while childhood cigarette smoke exposure was associated with increased risk of surgery (OR 1.82, 95% CI 1.14 – 2.91) (Table 2). Exposure to pets, growing up on a farm, or daycare exposure did not modify risk of surgery in CD. Among disease related covariates, as expected, older age at diagnosis was associated with reduced risk of surgery (OR 0.97, 95% CI 0.96 – 0.99) while both stricturing (OR 5.56, 95% CI 2.78 – 11.10) and penetrating phenotypes (OR 5.95, 95% CI 3.28 – 10.79) were associated with increased risk of surgery. On multivariate analysis, adjusting for age at diagnosis, disease duration, location, behavior, presence of perianal disease, and current smoking status, a history of being breastfed (OR 0.21, 95% CI 0.09 – 0.46) remained inversely associated while early life cigarette smoke exposure (OR 2.17, 95% CI 1.10 – 4.29) was associated with increased risk of surgery (Table 2). In a mutually adjusted model including both breastfeeding and childhood cigarette smoke exposure, only breast feeding remaining statistically significant in its inverse association with CD related surgery (OR 0.22, 95% CI 0.10 – 0.49). In exploratory analysis, neither a history of being breast fed nor early life cigarette smoke exposure was associated with anti-TNF use (a history of being breastfed: OR 1.06, 95% CI 0.59 – 1.93; childhood cigarette smoke exposure OR 0.75, 95% CI 0.42 – 1.33), penetrating or stricturing CD, with a specific disease location, or age at diagnosis.

**Table 2:** **Association between early life environmental exposures and surgery in Crohn’s disease**

**Exposure**	**Univariate OR**	**Multivariate** ^**+**^ **OR‖**
	**(95% CI)**	**(95% CI)**
**Breastfeeding**	0.42 (0.25-0.68)	0.21 (0.09 – 0.46)
**Exposure to cigarette smoke**	1.82 (1.14-2.91)	2.17 (1.10 – 4.29)
**Exposure to pets**	0.93 (0.58-1.50)	
**Growing up on farm**	1.30 (0.37-4.59)	
**Daycare exposure**	0.79 (0.49-1.24)	
**Delivery via caesarean section**	1.02 (0.53 – 1.98)	
**Hospitalization before age 5**	1.24 (0.70 – 2.19)	

^+^Adjusted for age, disease location (ileal, colon, ileocolon); phenotype (inflammatory, stricturing, penetrating), perianal involvement, duration of disease, and current smoking status (never, past, current).

‖Only variables significant for univariate analysis were further explored for multivariate analysis. Multivariate analysis included 249 patients in Crohn’s disease with non-missing data for all variables.

In contrast to the association for CD, we identified no association between early life exposures and need for surgery or biologic therapy in UC (Table 3). Such exposures were also not associated with disease extent except for a weak association between childhood cigarette smoke exposure and pancolitis (OR 3.25, 95% CI 1.02 – 10.34).

**Table 3:** **Association between early life environmental exposures and surgery in ulcerative colitis**

**Exposure**	**Univariate odds ratio‖**	**95% confidence interval**
**Breastfeeding**	1.51	0.63-3.61
**Exposure to cigarette smoke**	1.28	0.60-2.71
**Pets during childhood**	0.99	0.45-2.17
**Daycare**	1.96	0.92-4.18
**Hospitalized before age 5 years**	1.09	0.38-3.11
**Delivery via caesarean section**	0.63	0.18 – 2.22

‖As none of the variables were significant on univariate analysis, we did not perform multivariate adjusted analyses.

There was no IBD-related surgery among the 10 UC patients who reported growing up on a farm. Consequently, an odds ratio couldn’t be estimated.

## Discussion

Growing literature on genetics and the microbiome supported by data from prospective epidemiologic cohorts suggests an important role for the external environment in incidence and natural history of IBD [1,7,11]. Identification of relevant environmental exposures offer the promise of providing clues towards the etiopathogenesis of these complex and disabling diseases, and importantly allow for development of interventions that modify these factors to improve disease outcomes. This study, for the first time examined the association between early life exposures and disease outcome in IBD. Our analysis showed that a history of being breastfed was inversely associated with risk of surgery in CD, while exposure to cigarette smoke during childhood increased this risk independent of current smoking status.

The role of breastfeeding in immunologically mediated diseases has been examined in several prior studies; most have focused on it as a risk factor for incident disease and not on subsequent natural history. The reduction in risk of IBD in those who were breastfed in infancy was proposed in support of the hygiene hypothesis, which linked the rise in incidence of autoimmune disease to improvement in environmental hygiene and reduced exposure to childhood infections [24]. However, the association between a history of being breastfed and risk of incident CD or UC has been inconsistent in the literature. Several studies failed to identify such an association [16,25-27] while a Danish cohort suggested a trend towards protective effect [12]. A large population-based case control study by Gearry *et al*. including over 600 patients each with CD, UC, and healthy controls demonstrated a protective association between breastfeeding and both diseases [28]. Indeed, Corrao *et al*. demonstrated that lack of breastfeeding during infancy provided the highest population attributable risk for CD in women [29].

Newly established inception cohorts of CD confirm dysbiosis early on in disease [6]. Prior studies have shown that specific bacteria such as the firmicute *Faecalibacterium prausnitzii* may be protective against recurrence after surgical resection confirming the central role of enteric microbiota in the development of disease [9]. No prior studies examined the effect of breastfeeding on course of IBD. However, considerable biological plausibility exists to this hypothesis. Breastfeeding exerts one of the strongest influences on the gut microbiome. In an elegant study, Penders *et al*. sequenced the microbiome of 1,032 infants in the Netherlands and found that those who were exclusively breastfed had reduced colonization with *Clostridium difficile*, *Bacteroides*, and *Lactobacilli* and higher frequency of the protective bifidobacteria [30]. Other studies demonstrated similar changes in the enteric microbiome of breastfed infants [31,32]. This has mechanistic implications for the findings of our present study as specific microbial colonization in infancy such as with *C difficile* has been associated with increased risk of allergy and atopic disorders [30,33,34]. Thus it is plausible that early life factors that influence the establishment of the ‘normal’ microbiome may have persistent effects on immune reactivity, thereby also influencing natural history of chronic inflammatory diseases.

Considerable literature exists regarding the effect of cigarette smoke on CD and UC [11,35-38]. It is well recognized that current smoking is a risk factor for incident CD and more aggressive disease course [29,36-40]. Passive exposure to cigarette smoke has a similar direction of effect though the magnitude of association is weaker than that existing for personal history of smoking [41]. Our findings suggest that remote exposure even early on in childhood may exert a similar effect on natural history of CD. Several prior studies have suggested childhood exposure to be a risk factor for incident CD or UC [42,43] but have not examined it as a risk factor for more aggressive subsequent course. There are a few possible ways through which early exposure to cigarette smoke may exert its effect. First, it may alter the intestinal microbiome composition [44]. It has been consistently demonstrated that early life exposure may have greater and more sustained effects on the gut microbiome than exposures later on in adult life [30,33,34,45]. Second, cigarette smoke may disrupt protective immune mechanisms by impairing recovery from oxidative stress through its effect on the mononuclear cells [46]. Finally, it is possible that smoking may lead to epigenetic alterations that subsequently influence natural history of disease [47]. All these avenues merit further exploration and may offer important clues to the effect of environment on disease.

There are a few implications of our findings. To our knowledge, it is one of the first studies to demonstrate an association between specific early life environmental influences and subsequent outcomes in CD. The large size of our cohort with detailed exposure and outcome information allowed for sufficient power to test our hypotheses. If confirmed in subsequent cohorts, such studies can offer intriguing clues to pathogenesis and highlight the role of the differentially distributed microbiome species as potentially modifiable targets to improve disease outcomes. Further studies are required to prospectively sequence the microbiome at diagnosis in patients with established disease, ascertain environmental exposures, and examine the association with subsequent disease course. Research is also necessary to examine if this is conditional on host genotype as genetics may influence susceptibility to the environment.

We readily acknowledge several limitations to our study. First, exposure to early life environmental influences was through self-report. However, it is unlikely that self-report of early life risk factors would be susceptible to recall bias. Recall bias owing to differential time since diagnosis is also unlikely as the duration of disease was similar between both groups, and adjusted for in our final multivariate models. Second, we did not have information on duration of breastfeeding, exclusivity of breastfeeding, or age at weaning. Such factors may modify the association between breastfeeding and disease outcomes and merit further exploration. Third, residual confounding is a possibility as in any observational study. Fourth, due to fewer events (surgery) in UC, the statistical power to detect associations may be lower. However, most associations except for daycare exposure did not demonstrate even a trend towards an association. We also did not have information on other factors that have been associated with aggressive disease course including steroid use at diagnosis.

## Conclusions

In conclusion, this study identified a history of being breastfed and exposure to cigarette smoke during childhood as environmental factors that may impact the natural course of CD. Further studies that examine these factors in conjunction with changes in the microbiome, epigenetic changes, and gene expression could offer insight into how the natural history of IBD is influenced by such influences, and offer avenues for improving disease outcomes.

## Additional file

Additional file 1:
**Questionnaire ascertaining early life environmental exposures.**
