# Supplementary material for: Early life environment and natural history of inflammatory bowel diseases
Source: BMC Gastroenterol. 2014 Dec 16;14:216. doi: 10.1186/s12876-014-0216-8 (PMC4300207; doi:10.1186/s12876-014-0216-8)
Supplement: Additional file 1: — Questionnaire ascertaining early life environmental exposures. [file 12876_2014_216_MOESM1_ESM.doc]

**Additional file 1: Questionnaire ascertaining early life environmental exposures**

**Adult Baseline Environmental Questionnaire**

**Early Childhood:**

1) Were you born in a hospital?

Yes No Not sure

2) Were you born via C-section?

Yes No Not sure

3) Were you born in the United States?

Yes No Not sure

If no, where were you born? __________________________________

If no, in what year did you come to the United States?______________

4) Were you breastfed as an infant?

Yes No Not sure

5) Were you treated with antibiotics before the age of one?

Yes No Not sure

6) Were you hospitalized before the age of 5?

Yes No Not sure

7) Did you have pets growing up?

Yes No Not sure

8) Did you grow up on a farm?

Yes No Not sure

9) Did you attend daycare as a child?

Yes No Not sure

10) Were you exposed to cigarette smoke as a child?

Yes No Not sure

11) Were you born prematurely (more than 3 weeks early)?

Yes No Not sure
